# The Mystery of EVP4593: Perspectives of the Quinazoline-Derived Compound in the Treatment of Huntington’s Disease and Other Human Pathologies

**DOI:** 10.3390/ijms232415724

**Published:** 2022-12-11

**Authors:** Dmitriy A. Grekhnev, Anna A. Kruchinina, Vladimir A. Vigont, Elena V. Kaznacheyeva

**Affiliations:** Laboratory of Ionic Channels of Cell Membranes, Department of Molecular Physiology of the Cell, Institute of Cytology, Russian Academy of Sciences, 4 Tikhoretsky Ave., St. Petersburg 194064, Russia

**Keywords:** quinazoline derivatives, QNZ, EVP4593, calcium signaling, NF-κB signaling, store-operated calcium entry, Huntington’s disease, huntingtin, neurodegeneration, oncology

## Abstract

Quinazoline derivatives have various pharmacological activities and are widely used in clinical practice. Here, we reviewed the proposed mechanisms of the physiological activity of the quinazoline derivative EVP4593 and perspectives for its clinical implication. We summarized the accumulated data about EVP4593 and focused on its activities in different models of Huntington’s disease (HD), including patient-specific iPSCs-based neurons. To make a deeper insight into its neuroprotective role in HD treatment, we discussed the ability of EVP4593 to modulate calcium signaling and reduce the level of the huntingtin protein. Moreover, we described possible protective effects of EVP4593 in other pathologies, such as oncology, cardiovascular diseases and parasite invasion. We hope that comprehensive analyses of the molecular mechanisms of EVP4593 activity will allow for the expansion of the scope of the EVP4593 application.

## 1. Introduction

Quinazoline derivatives are a large pool of natural and synthetic compounds. The first derivatives of quinazoline were synthesized at the end of the 19th century [1]. To date, more than 200 derivatives of quinazoline with various biological activities, including anticancer, antihypertensive, antiviral, antimicrobial, antifungal, antiparasitic, anti-inflammatory and anticonvulsant, are known [2]. Many quinazoline derivatives have low toxicity and are widely used in clinical treatment [3]. Nevertheless, the mechanisms of pharmacological activities of many quinazoline derivatives remain poorly understood. Among the key targets of quinazoline derivatives are kinases, transcription factors, receptors and ion channels [3,4,5,6,7]. Here, we focused on one quinazoline derivative (4-N-[2-(4-phenoxyphenyl)ethyl]quinazoline-4,6-diamine)—EVP4593 (also marked as QNZ in papers). This compound was originally synthesized in 2003 as a modulator of the nuclear factor kappa B (NF-κB) signal transduction pathway [8,9]. Since that time, EVP4593 has been widely used as a blocker of NF-κB signaling (Sigma-Aldrich, cat #481417). Then, it has been postulated that EVP4593 affects calcium entry through store-operated calcium (SOC) channels [10], which are well-known to be required for the initiation of NF-κB signaling [11,12,13].

Firstly, the ability of EVP4593 to reduce store-operated calcium entry (SOCE) and protect neurons was shown in human neuroblastoma SK-N-SH cells, modeling Huntington’s disease (HD) [10]. In this review, we analyzed possible molecular mechanisms of EVP4593 activity and summarized the experience of using this compound in the treatment of various human pathologies.

## 2. The Molecular Mechanisms of EVP4593 Activity

### 2.1. EVP4593 and NF-κB Signaling

**Figure 1:**
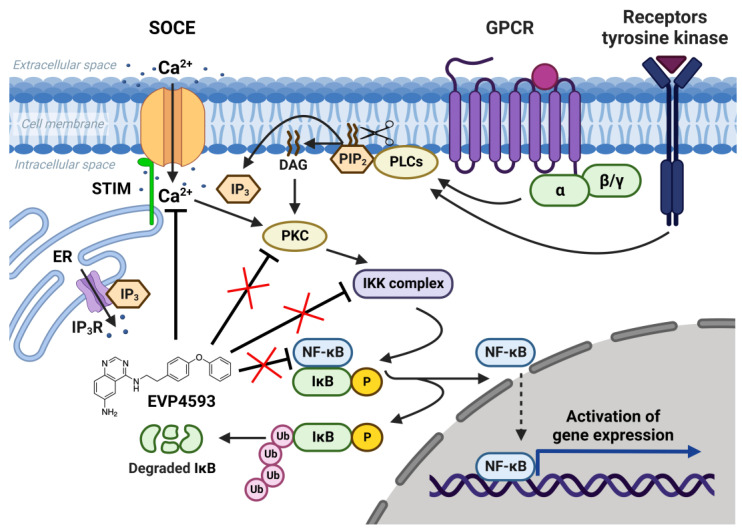
NF-κB signaling pathway and SOCE. SOC channels are activated as a result of intracellular calcium store depletion caused by the activation of inositol-1,4,5-trisphosphate receptor (IP_3_R). Calcium influx through SOC channels activates protein kinase C (PKC), which activates the IκB kinase (IKK) complex containing the kinases IKKα and IKKβ and the regulatory protein IKKγ. Activated IKK complex phosphorylates inhibitory protein IκB and recruits NF-κB dimers (p50, p52, p65/RelA, RelB and c-Rel). NF-κB free from inhibitory protein IκB migrate to the nucleus and initiate gene expression. EVP4593 does not directly affect any key protein of the NF-κB signal pathway but inhibits SOCE, which is required for NF-κB activation.

The NF-κB signaling pathway is one of the general signal transduction pathways that mediate gene expression of proinflammatory and antiapoptotic factors, cell proliferation, differentiation, adhesion, migration and angiogenesis [14]. The hyperactivation of NF-κB signaling is a hallmark of many types of cancer [14] and inflammatory-associated pathologies [14,15]. Additionally, the chronic inflammation associated with upregulation of NF-κB signaling is a common feature of multiple neurodegenerative diseases [16]. Thus, the search for selective blockers of NF-κB signaling is an attractive task for both anticancer and anti-neurodegenerative disorders’ drug discovery. In 2003, Tobe et al. synthesized a novel pool of quinazoline derivatives that could act as antagonists of the NF-κB signaling pathway [8,9]. Analyzing various quinazoline core derivatives, Tobe et al. revealed the most effective compound, EVP4593 (4-N-[2-(4-phenoxyphenyl)ethyl]quinazoline-4,6-diamine), blocking NF-κB signaling at nanomolar concentrations (IC_50_ 11 nM) [9]. However, even almost 20 years later, the specific molecular target for EVP4593 remains unclear. It has been established that the EVP4593 does not directly influence any key protein involved in NF-κB signaling, including protein kinase C (PKC), IκB kinase (IKK) complex and transcriptional factor NF-κB (Figure 1) [10]. Therefore, it was assumed that the molecular target of the EVP4593 should be located upstream of NF-κB signaling. The key upstream activators of NF-κB signaling are receptors of interleukins, tumor necrosis factor, lipopolysaccharides and calcium ions [14]. It is well-known that calcium influx through SOC channels triggers the activation of the NF-κB cascade [9,10,11] (Figure 1). Moreover, established SOCE blockers SKF96365 [17] and BTP2 [18] were reported to suppress NF-κB activity. The hypothesis that EVP4593 affects SOC channels, thus modulating NF-κB signaling, was proven for the first time in 2011 by Wu et al. [10].

### 2.2. EVP4593 and SOCE

In 2011, it was shown for the first time that EVP4593 at a concentration of 300 nM can reversibly block SOC channels and reduce pathologically elevated SOCE in an HD neuroblastoma cell model, expressing full-length mutant huntingtin (138Q), and in striatal neurons isolated from HD-specific YAC128 mice [10]. The current–voltage relationships of the registered SOC currents corresponded to the SOC channels formed by the transient receptor potential canonical (TRPC) proteins [10]. Moreover, suppression of TRPC1 significantly reduced SOC currents in SK-N-SH cells, modeling HD; at that point, the remaining currents were almost insensitive to further inhibition by EVP4593. Nevertheless, EVP4593 failed to inhibit SOC currents in SK-N-SH cells with overexpressed TRPC1 [10]. Therefore, it was concluded that EVP4593 affects heteromeric channels containing TRPC1 as one of the subunits but not homomeric TRPC1 channels. Besides SOC channels’ inhibitory activity, EVP4593 was shown to delay a progression of a motor phenotype in the fly model of HD [10]. Thus, SOC channels (especially TRPC1-containing) have become a potential molecular target for EVP4593, and EVP4593 was established as a promising pharmacological substance for HD treatment.

**Figure 2:**
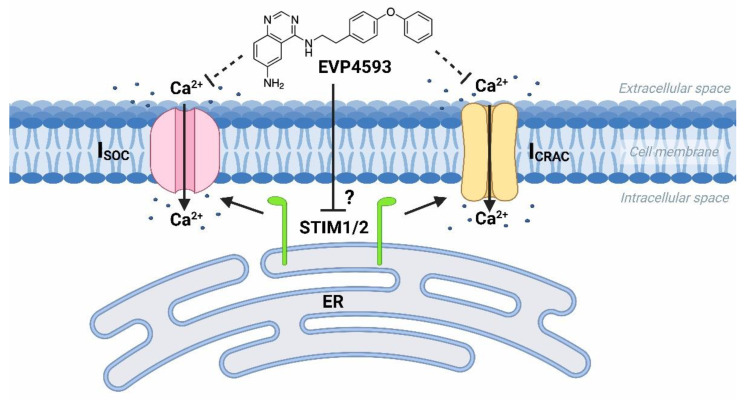
EVP4593 inhibits both Orai- and TRPC-contained store-operated calcium channels. EVP4593 blocks currents through both types of SOC channels that are Orai-contained (I_CRAC_) and TRPC-contained (I_SOC_).

At the same time, the future studies of HD-specific induced pluripotent stem cells (iPSCs)-based GABAergic medium spiny neurons (MSNs) obtained from adult onset HD patients (40–47 glutamine residues in the polyglutamine tract of mutant huntingtin) demonstrated that both Orai- and TRPC-contained SOC channels were sensitive to treatment by 100 nM EVP4593 [19]. Thus, it has been supposed that the molecular target for EVP4593 may represent a common regulator of SOC channels, such as STIM1 or STIM2 proteins (Figure 2).

**Figure 3:**
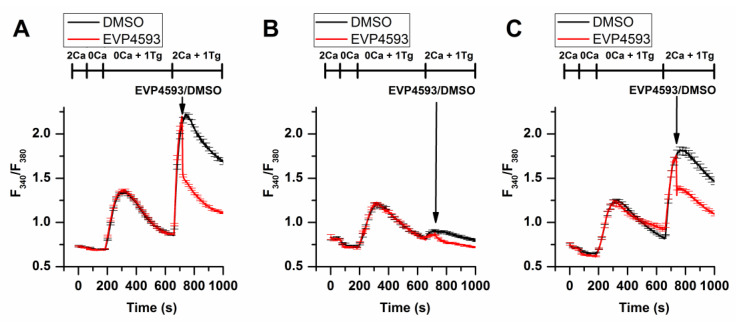
EVP4593 suppresses SOCE in both STIM1 (STIM1KO) and STIM2 (STIM2KO) knockout HEK293 cell lines. Normalized relative fluorescence of Fura-2AM associated with cytosolic calcium level in HEK293 (**A**), HEK293 STIM1KO (**B**) and HEK STIM2KO (**C**) cells during thapsigargin-induced calcium response. The arrow indicates the supply of 1 µM EVP4593 or 0.1% DMSO, respectively. The used solutions are indicated above the curves. The curves are plotted as the mean ± SEM.

Additional experimental data obtained by using fluorescent calcium imaging (the method’s description is available in [20]) indicated that EVP4593 could not discriminate between STIM1 and STIM2 in HEK293 cells, affecting SOC in both STIM1 and STIM2 knockout cell lines (Figure 3).

To summarize the results obtained and published data, we cannot unequivocally explain the inhibitory effect of EVP4593 on SOCE. So, despite the low effective concentration of EVP4593, which supposes an existing direct protein target, we cannot exclude affecting SOC channels through the modulation of plasma membrane features.

### 2.3. EVP4593 and Mitochondrial Complex I

In addition to the well-studied effects of EVP4593 on SOCE and NF-κB signaling, Robin Krishnathas and colleagues identified mitochondrial complex I as a novel target for EVP4593 [21]. EVP4593 has been shown to exhibit strong inhibitory activity against mitochondrial complex I at nanomolar concentration. Molecular docking predictions illustrated that EVP4593 may incorporate into the quinone binding site, thus inhibiting mitochondrial complex I [22]. However, EVP4593 targeting mitochondrial complex I did not explain the inhibitory effect on SOCE and NF-κB signaling. Perhaps there are some independent targets for EVP4593. It should be noted that the velocity of the EVP4593 effects detected in experiments does not unequivocally conclude which target is primary from between mitochondrial complex I or SOC channels.

Mitochondrial complex I dysfunctions are also associated with oncogenesis and some neurodegenerative disorders [23,24,25]. Thus, EVP4593 can be successfully used in the treatment of cancer and neurodegenerative diseases, with a suitable overlap in effects on SOCE and mitochondrial complex I.

### 2.4. EVP4593 and mTOR Signaling

mTOR (Mechanistic/Mammalian Target of Rapamycin) signaling controls a wide range of processes involved in cellular metabolism and plays a crucial role in autophagy regulation [26,27]. Aberrant mTOR signaling is associated with a number of pathologies. Hyperactivated mTOR signaling marks some types of cancer and promotes proliferation and tumor progression [26]. mTOR signaling inhibitors are considered promising anticancer drugs [28]. Indeed, the promising therapeutic strategy is the suppression of the mTOR signaling pathway and, consequently, the enhancement of autophagy [27]. Ran Marciano, Manu Prasad and colleagues firstly found that EVP4593 inhibited the mTOR pathway in tumor cells growing under glucose starvation but not under normal conditions [29]. This fact indicates the absence of direct interaction of EVP4593 with the components of mTOR signaling. At the same time, low levels of phosphorylated-4EBP1, phosphorylated-P70S6K and phosphorylated-S6RP under EVP4593 treatment indicates inhibition of the mTORC1 pathway [29]. Since EVP4593 may inhibit mitochondrial complex I, the tumor cells pretreated by EVP4593 may have a significantly lower level of ATP under glucose deprivation, which leads to an inhibition of the AMPK/mTORC1 pathway and suppression of tumor progression. Additionally, EVP4593 inhibits the NF-ĸB pathway that promotes survival under glucose starvation (as well as other NF-ĸB inhibitors) [30]. Thus, EVP4593 promotes the selective death of the most glucose-dependent tumor cells.

The upregulation and involvement of the mTOR signaling pathway in neurodegenerative pathologies, including Huntington’s disease, is excellently discussed in the review by Professor Henry Querfurth [27]. The autophagy induction by mTOR signaling antagonists is accompanied by the removal of mutant protein aggregates and may protect neurons from degeneration [27]. Previously, we have reported larger numbers of lysosomes and autophagosomes in patient-specific models of HD compared to control healthy donors [31], supposing a compensatory enhancement of autophagy in HD-specific neurons aimed at cell survival. Since EVP4593 was shown to inhibit mTOR signaling in cancer [29], we anticipated the induction of autophagy upon EVP4593 treatment. However, HD-specific MSNs (but not WT MSNs) demonstrated a decreased number of lysosomes/autophagosomes after the EVP4593 application [31]. Moreover, it was also reported that other NF-κB inhibition may reduce the number of autophagosomes [32]. Possible controversies can be potentially solved because the reduced number of autophagosomes may indicate the high efficacy of autophagy, whereas a large number of autophagosomes may be a result of autophagy block and the accumulation of “pre-autophagosomes”.

**Figure 4:**
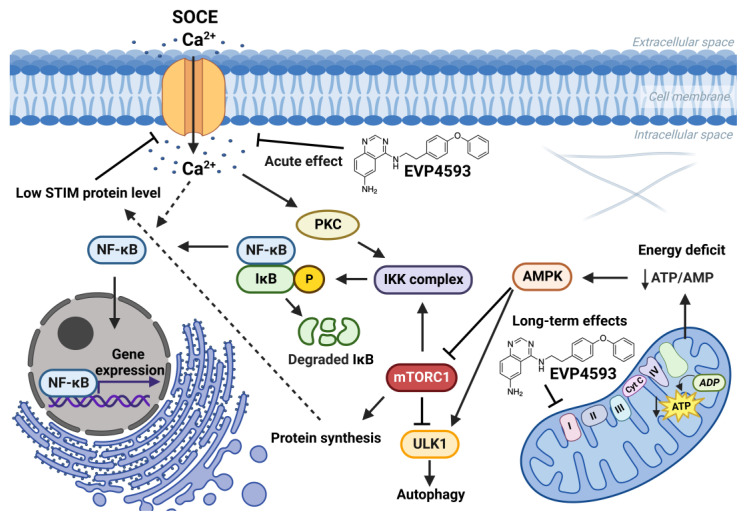
Effects of EVP4593 on cell signaling. EVP4593 has both acute and long-term effects. The acute effect is to directly inhibit SOCE. Long-term effects may be associated with the inhibition of the mitochondrial complex I. Energy deficit induced by EVP4593 inhibits the AMPK/mTORC1 pathway or activates Unc-51 such as autophagy activating kinase 1 (ULK1), which leads to enhanced autophagy and suppressed protein synthesis. Additionally, EVP4593 can negatively affect NF-κB signaling by inhibiting SOCE or mTORC1 signaling. Consequently, EVP4593 also suppresses the expression of NF-κB -dependent genes. The lower levels of STIM proteins due to EVP4593 action may also result in reduced SOCE.

The connection between mTORC1 signaling and SOCE is interesting. The data suggest that mTORC1 signaling is a positive regulator of SOCE [33,34,35]. Additionally, SOCE inhibition led to enhanced autophagy probably through the Akt/mTOR signaling pathway [36,37]. Furthermore, mTORC1 is a positive regulator of NF-κB signaling [38]. mTORC1 may phosphorylate IKKα/β and induce the transcriptional activity of NF-κB [38]. Resveratrol, which is known to inhibit NF-κB and mTOR signaling, reduce SOCE in preincubation experiments and also enhance autophagy [39]. However, the role of calcium signaling in the regulation of autophagy remains complicated [40]. Therefore, for the long-term effect of EVP4593, the following axis can be formed: inhibition of mitochondrial complex I—inhibition of the AMPK/mTORC1 pathway (or Akt/mTOR pathway)—inhibition NF-κB signaling—inhibition of SOCE (Figure 4). Nevertheless, EVP4593 also demonstrates an acute inhibitory SOCE effect confirmed by patch-clamp experiments and fluorescent calcium imaging [10,19,31,41] (Figure 3 and Figure 4).

### 2.5. EVP4593 and Gene Expression

**Figure 5:**
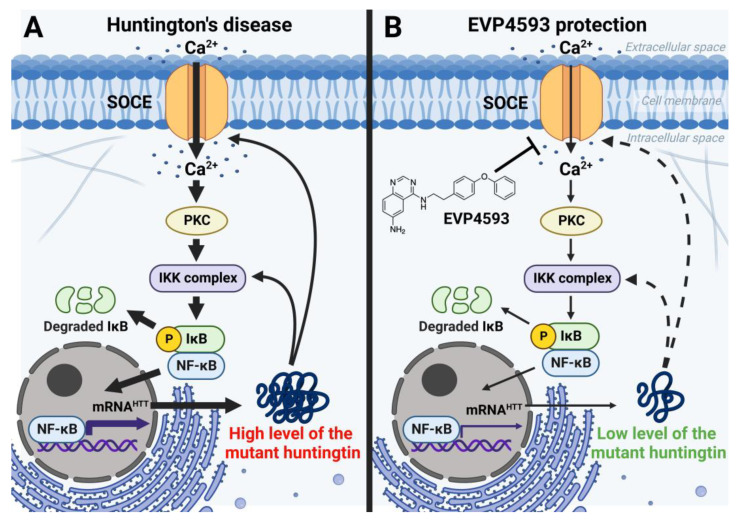
EVP4593 reduces the level of the huntingtin protein. Huntingtin gene expression is regulated by NF-κB-dependent promoter/enhancer. (**A**) Elevated SOCE in HD induces hyperactivation of NF-κB signaling, resulting in a high level of the huntingtin protein. Mutant huntingtin enhances calcium influx through SOC channels and potentiates the IKK complex. Thus, a pathological vicious circle is formed. (**B**) EVP4593 attenuates pathologically enhanced SOCE and decreases NF-κB-dependent huntingtin production.

In addition to an acute inhibitory effect of EVP4593 on SOCE, the long-term effects of incubation cells with EVP4593 have also been observed. The modulation of gene expression by EVP4593 is obvious since the expression of many genes depends on NF-κB. Indeed, it was found that incubation of HD-specific MSNs with 300 nM EVP4593 for 24–48 h reduced excessive levels of the huntingtin protein [42]. These data were not surprising because the expression of the huntingtin gene depends on NF-κB [43] (Figure 5). Notably, modulation of huntingtin expression may have therapeutic applications. It has been shown that even a 10% decrease in mutant huntingtin level has a neuroprotective effect, while even a 90% decrease in normal huntingtin level has no pathological effect [44].

What is interesting is that proteins, encoded by NF-κB-independent genes, may also change their levels upon treatment by EVP4593. SOC channels’ activator STIM2, which has a high level associated with excessive SOCE in HD-specific neurons [42], can also be downregulated by the application of EVP4593, thus protecting neurons from toxic calcium influx. Another research group reported that excessive STIM2-dependent SOC channels’ activity appears to lead to spine loss in YAC128 (HD mice model) MSN [45], confirming the key role of STIM2 at a high level in HD pathogenesis and establishing STIM2 as a promising target for anti-HD drugs. On the other hand, it is extremely important that EVP4593 reduces excessive STIM2 levels to control values since it has been reported that downregulation of STIM2 can be dangerous because STIM2-dependent stability of mushroom spines was shown to be a mechanism of hippocampal synaptic loss in a mice model of Alzheimer’s disease [46]. Curiously, despite the expression of the STIM2 encoding gene, it does not depend on NF-κB; another blocker of NF-κB signaling, wogonin, can also reduce STIM2 levels [47]. Wogonin also inhibits the mTOR pathway and can have a similar effect as rapamycin on the STIM protein level [48].

## 3. Perspectives of EVP4593 in Clinical Trials

### 3.1. Neurodegeneration

Impairment of calcium signaling and neuroinflammation are the key features accompanying various neurodegenerative pathologies [49,50,51,52]. Correction of pathological calcium signaling, including SOCE and suppression of the NF-κB signaling pathway, has a neuroprotective effect and can prevent the development of pathological processes [10,31,52,53,54]. Here, we summarized the reported effects of EVP4593 on various models of human diseases (Table 1).

It emphasized the low toxicity of EVP4593 and its pronounced neuroprotective effect in different HD models, including mice [46] and fly models [10]. Moreover, the majority of studies indicate that EVP4593 is effective at high nanomolar concentrations, which makes it suitable for further clinical implications. The main question is, what are the reasons for the neuroprotective effect of EVP4593? Is the observed neuroprotection associated with the inhibition of the NF-κB pathway or mitochondrial complex I or it is a consequence of the SOCE reduction? It was shown that EVP4593-mediated inhibition of the NF-κB pathway is probably not the main cause of the neuroprotective effect observed in experiments with transgenic HD flies [10]. Additionally, the structurally unrelated inhibitor of IKK and NF-κB pathway BMS-345541 were not effective in the fly HD model [10]. Thus, the suppression of NF-κB signaling is probably a necessary but not sufficient condition for neuroprotective effect in HD.

In the case of HD, we can speculate about the key effect of EVP4593 to attenuate excessive huntingtin level. Since HD is a monogenic disorder, all alterations in cellular processes are obviously caused by the presence of the toxic mutant huntingtin. However, EVP4593 cannot completely eliminate mutant huntingtin from the cells. Moreover, it should be noted that despite huntingtin expression being ubiquitous, many neurodegenerative pathologies, including HD, are characterized by selective death of specific types of neurons. Indeed, the most affected neurons in HD are MSNs [55], whereas Parkinson’s disease is characterized by predominant degeneration of dopaminergic neurons in the substantia nigra [56], and significant loss of hippocampal neurons is observed in Alzheimer’s disease [57]. Trying to develop the previously established calcium hypothesis of neurodegeneration [58], we suggest formulating “The calcium hypothesis of selective neuronal death”, where the selective vulnerability of neurons can be determined by different patterns of calcium signaling alterations and different potentials of the neurons to compensate them (for example, by the different levels of calcium-binding proteins). The accumulated data indicated that improper functioning of calcium channels, including SOC channels, plays a crucial role in a number of neurodegenerative pathologies [50,54,59]. Thus, the ability of EVP4593 to reduce excessive SOCE opens up perspectives to develop novel neuroprotective drugs based on EVP4593 for the selective treatment of neurodegenerative disorders. It also requires future studying of potentially selective effects of EVP4593 on different neuronal types.

### 3.2. Other Pathologies

EVP4593 has been also established as an antineoplastic compound that can suppress carcinogenesis [60,61]. In various carcinoma cell lines, EVP4593 exhibits strong antitumor effects (Table 1). To sum up, EVP4593 suppresses uncontrolled cell proliferation and migration, inhibits expression of the antiapoptotic proteins and increases the level of the proapoptotic proteins [60,61,62,63,64]. Noted antitumor effects of EVP4593 make it to become a prospective anticancer drug.

Another area of potential EVP4593 implication is cardiovascular pathologies. These aspects of EVP4593 actioning are studied poorly. Nevertheless, the ability of EVP4593 to enhance morphine-induced cardio protection in a myocardial ischemia/reperfusion rat model was shown [64]. Moreover, SOCE was registered in adult ventricular cardiomyocytes isolated from mice [65], suggesting its physiological role in the functioning of the cardiac cells. So, the ability of SOCE modulators to correct aberrant calcium signaling in cardiomyocytes may be in demand soon.

Finally, NF-kB is a key inflammatory mediator that regulates the production of many proinflammatory factors, including NO, which plays a central role in the elimination of parasitic infestations. Notably, the published data demonstrated the anthelmintic [66] and antileishmanial [67] activities of EVP4593 further expand the range of its possible implication.

**Table 1:** The physiological effects of EVP4593 in various models of human diseases.

Model	Mechanism of EVP4593 Activity	EVP4593 Concentration	Potential Treatment	References
Neurodegeneration				
Juvenile and adult-onset HD iPSCs-derived GABA neurons	EVP4593 attenuates pathologically enhanced huntingtin and STIM2 level. Pretreated-by-EVP4593 HD-specific neurons demonstrate reduced SOCE.	300 nM	HD	[42]
Adult-onset HD iPSCs-derived GABA neurons	EVP4593 inhibits both I_CRAC_ and I_SOC_ channels.	100 nM	HD	[19]
Adult-onset HD iPSCs-derived GABA neurons	EVP4593 attenuates SOCE both in wild-type and HD-specific neurons. EVP4593 reduces the number of lysosomes/ autophagosomes. EVP4593 reduces MG132-induced HD-specific neuronal death.	100 nM	HD	[31]
The primary culture of mice MSNs with expressed Htt138Q-1exon	EVP4593 reduces SOCE to the normal level.	300 nM	HD	[41]
YAC128 transgenic HD mice. Mixed cortical/striatal (MSNs) cultures	EVP4593 reduces synaptic neuronal SOCE and rescues spine loss. Intraventricular delivery of EVP4593 in YAC128 mice rescues age-dependent striatal spine loss in vivo.	30 nM and 0.25 mg/mL in vivo	HD	[45]
YAC128 transgenic HD mice. The primary culture of mice MSNs. Fly HD model (*Drosophila melanogaster*) with expressed first 4 exons of human huntingtin (128Q)	EVP4593 attenuates enhanced SOCE. EVP4593 affects heteromeric channels containing the TRPC1 subunit but has no effect on homooligomer channels composed of TRPC1. EVP4593 delays a progression of a motor dysfunction phenotype in a transgenic fly HD model and protects YAC128 MSNs in a glutamate toxicity assay.	300 nM and 100–400 µM in vivo	HD	[10]
R6/2 transgenic HD mice. The primary culture of cortical pyramidal neurons	EVP4593 reduces somatic calcium transient oscillations.	3 µM	HD	[68]
The primary hippocampal cultures obtained from mice with PSEN1ΔE9 expression	EVP4593 decreases PSEN1ΔE9-mediated SOCE and rescues mushroom spines in PSEN1ΔE9-expressing neurons.	30 nM	AD	[46]
Oncology				
Hepatoblastoma cell line (HepG2) and hepatocellular carcinoma cell lines (Huh7 and SNU-387)	EVP4593 affects NF-κB signaling and has an antitumor effect.	Huh7 and SNU-387—5 µM and HepG2—10 µM	Liver cancer	[60]
Head and neck squamous cell carcinoma (HNSCC) cell lines PCI1, PCI9, PCI13 and PCI52	EVP4593 inhibits the NF-κB signaling. Antitumor effects.	1–10 μM	Cancer	[61]
Breast cancer cell lineMCF7 (Michigan Cancer Foundation-7)	EVP4593 inhibits the NF-κB signaling and the mTOR pathway. Antitumor effects.	1 mg/kg	Cancer	[29]
Nonsmall cell lung cancer (NSCLC) CL1-5-F4 cells	EVP4593 suppresses the NF-ĸB activation. EVP4593 inhibited the expression of metastasis-associated proteins. EVP4593 reduced cell migration and invasion.	0.25 μM	Lung cancer	[62]
SK-HEP-1 cells	EVP4593 significantly inhibits the expression of antiapoptotic proteins and triggers extrinsic and intrinsic apoptosis pathways. EVP4593 reduces cell viability. EVP4593 inhibits the expression of NF-κB p65 and antiapoptotic proteins (XIAP, MCL-1 and c-FLIP) and increases levels of proapoptotic proteins (caspase-3 and -8 and cytochrome c).	0.4 µM	Human hepatocellular carcinoma	[63]
Parasites invasion				
*L.amazonensis*-infected macrophages	EVP4593 inhibits amastigote growth and induces the production of high levels of NO and IL-1β.	10 μM	Antileishmanial activity	[67]
L3 larvae of *C. oncophora*	Anthelmintic activity	1.9–3.4 μM	Anthelmintic activity	[66]
Cardiovascular diseases				
Myocardial ischemia/reperfusion rat model	EVP4593 inhibits the NF-κB pathway, decreases the expression of NF-κB and has an anti-inflammatory effect. EVP4593 enhances morphine-induced cardio protection.	1 mg/kg	Myocardial infarction	[64]
Diabetic retinopathy				
Streptozotocin-induced diabetes model rats	EVP4593 could alleviate the aggravation of retinopathy. EVP4593 decrease the endothelial cell proliferation and significantly reduces p65 expression. EVP4593 reduces blood glucose level.	80 mg/kg	Diabetic retinopathy	[69]

## 4. Conclusions

To date, EVP4593 is a widely used inhibitor of NF-κB signaling. It is also established as an effective blocker of SOCE, which is probably associated with the suppression of NF-κB activity. However, it is still confusing to unequivocally explain the molecular mechanism of EVP4593 acute effects on SOC channels. Nevertheless, the repeatedly demonstrated protective effects of EVP4593 in various models of oncological and neurodegenerative disorders make it attractive for further clinical implications. Moreover, in the last decade, EVP4593 have become an important instrument for studies of the NF-κB signaling and SOC channels.

The multiple actions described for EVP4593 at first glance complicate its application for the development of selective therapy. However, today, the polypharmacological approach has become more and more popular, in particular, for the treatment of multifactorial pathologies such as neurodegenerative diseases [70]. Thus, the ability of EVP4593 to modulate NF-κB signaling, calcium signaling, mitochondrial activity, mTOR pathway and, consequently, autophagy makes it attractive for the development of a polyfunctional drug, generally affecting intracellular signaling and targeting overall cell survival. We hope that future investigations determine the direct molecular target for EVP4593 and expand the area of its clinical implication.

## Data Availability

Not applicable.
